# Visuo-Kinetic Signs Are Inherently Metonymic: How Embodied Metonymy Motivates Forms, Functions, and Schematic Patterns in Gesture

**DOI:** 10.3389/fpsyg.2019.00254

**Published:** 2019-02-27

**Authors:** Irene Mittelberg

**Affiliations:** Natural Media Lab, Center for Sign Language and Gesture and Institute of English, American and Romance Studies, RWTH Aachen University, Aachen, Germany

**Keywords:** gesture, metonymy, frames, scenes, iconicity, contiguity, indexicality, schematicity

## Abstract

This paper aims to evidence the inherently metonymic nature of co-speech gestures. Arguing that motivation in gesture involves iconicity (similarity), indexicality (contiguity), and habit (conventionality) to varying degrees, it demonstrates how a set of metonymic principles may lend a certain systematicity to experientially grounded processes of gestural abstraction and enaction. Introducing *visuo-kinetic signs* as an umbrella term for co-speech gestures and signed languages, the paper shows how a frame-based approach to gesture may integrate different cognitive/functional linguistic and semiotic accounts of metonymy (e.g., experiential domains, frame metonymy, contiguity, and pragmatic inferencing). The guiding assumption is that gestures metonymically profile deeply embodied, routinized aspects of familiar scenes, that is, the motivating context of frames. The discussion shows how gestures may evoke frame structures exhibiting varying degrees of groundedness, complexity, and schematicity: basic physical action and object frames; more complex frames; and highly abstract, complex frame structures. It thereby provides gestural evidence for the idea that metonymy is more basic and more directly experientially grounded than metaphor and thus often feeds into correlated metaphoric processes. Furthermore, the paper offers some initial insights into how metonymy also seems to induce the emergence of schematic patterns in gesture which may result from action-based and discourse-driven processes of habituation and conventionalization. It exemplifies how these forces may engender grammaticalization of a basic physical action into a gestural marker that shows strong metonymic form reduction, decreased transitivity, and interacting pragmatic functions. Finally, addressing basic metonymic operations in signed lexemes elucidates certain similarities regarding sign constitution in gesture and sign. English and German multimodal discourse data as well as German Sign Language (DGS) are drawn upon to illustrate the theoretical points of the paper. Overall, this paper presents a unified account of metonymy’s role in underpinning forms, functions, and patterns in visuo-kinetic signs.

## Introduction

Gestures are essentially metonymic: Iconic gestural figurations and enactments, in particular, exhibit the principle of partial semiotic portrayal par excellence. In interaction with concurrent speech, evanescent hand shapes and movements tend to abstract salient characteristics from, briefly allude to, or otherwise evoke entire persons, three-dimensional objects, holistic motion events, and rich contexts (e.g., Gibbs, 1994; Müller, 1998; Bouvet, 2001; Mittelberg, 2006; Calbris, 2011). With their gestures and postures, speakers typically foreground the particular aspects of previously witnessed or newly imagined objects, actions, behaviors, or scenarios that are especially relevant to their communicative intentions in ongoing discourses. They may trace, for instance, the spatial proportions of a building in the air or imitate a person’s action, such as running to catch a bus or handing a present to someone, in a reduced or stylized fashion. Interlocutors may thus, consciously or not, convey essential facets and kinesthetic qualities of their embodied experiences, memories, habits, mental imagery, or the immediate environment by schematically but effectively making them tangible and thus intersubjectively sharable in the here and now of a given multimodally orchestrated speech event (e.g., Sweetser, 2007; Mittelberg, 2013; Müller, 2014, 2017).

For example, if I am telling a friend that I will be spending the entire weekend working on my paper and simultaneously make a fleeting typing action, my hands simulate typing on an imaginary keyboard. From such a quick iconic gestural action, the addressee may infer that I will, in fact, be carefully and concentratedly typing for hours on the keyboard that actually exists on my desk. She can also infer the fact that, in this context, “working” means writing with the help of a computer. Moreover, she can imagine the written text that will result from this action, the content of the paper she knows I am working on, as well as other practically and ideationally related actions, entities, stages, versions, and mental or emotional states involved in eventually reaching the goal of submitting the finalized manuscript. All these various aspects are metonymically linked in a pragmatically structured context of experience, or *frame* (Fillmore, 1982), in which one gesture may evoke not only the immediately contiguous virtual keyboard, but also trigger an ensuing associative chain and a larger semantic network (e.g., Calbris, 2011; Mittelberg and Waugh, 2014; Mittelberg, 2017a).

### Metonymic Motivation of Gestural Abstraction and Enaction: More Than Iconicity

The primary aim of this paper is to pinpoint the inherently metonymic nature of co-speech gestures. It will show how distinct metonymic principles may lend a certain experientially grounded regularity to processes of *ad hoc* abstraction and enaction that are involved in gestural sign formation (e.g., Arnheim, 1969; Müller, 1998). Gestural abstraction and the resulting schematicity here are assumed not to be random, but experientially, cognitively, linguistically, pragmatically, and culturally motivated. Due to the temporal dynamics of face-to-face communication, there is only a very limited amount of time to perform gestures in sync with the conceptual contents, incremental articulation, and prosodic contours of the simultaneously evolving utterance as well as with other bodily signs such as gaze and head movements.

Crucially, visual perception, often somewhat privileged in cognitive approaches to language, is only one sensory, experiential source from which gesturers intuitively draw their semiotic material. A point the present proposal wishes to make is that motivation in gesture involves more than iconicity (e.g., McNeill, 1992; Mittelberg, 2006, 2014; Perniss et al., 2010).^1^ It is claimed that in gesture, besides interacting with iconicity and metaphoricity, it is through indexicality that metonymy also operates on latent contiguity relations between the hands and the material and social world.^2^ Such contiguity relations may become operationalized for gestural communication and thus lead the interpreting mind to ‘grasp’ the virtual objects and tools that gesturing hands seemingly manipulate (e.g., Müller, 1998; Streeck, 2009; Mittelberg and Waugh, 2014). By laying out how gestures may metonymically evoke frames through picking out aspects of *basic scenes of experience* (Fillmore, 1977, 1982; Goldberg, 1995, 1998), it will further be argued that a frame-based approach to gesture may not only integrate various accounts of metonymy (e.g., Dancygier and Sweetser, 2014), but also account for processes of pragmatic inferencing that are often heavily involved in gesture interpretation.

While the paper focuses on spontaneous gestures that are temporally, semantically, and syntactically integrated with concurrent speech (e.g., McNeill, 1992; Müller, 1998; Kendon, 2004; Fricke, 2012), metonymic modes underpinning iconic signs in sign language will also be addressed to highlight some commonalities regarding principles of sign constitution. Furthermore, the paper offers some initial insights into how embodied metonymic principles also seem to underpin discourse-pragmatic processes of routinization and schematization in gesture; that is, how metonymy may induce the emergence of gestural patterns with increased degrees of habit-driven conventionalization (e.g., Mittelberg, 2006, 2017a,c; Müller, 2017). Overall, this paper presents the first unified account of metonymy’s role in underpinning forms, functions, and patterns in visuo-kinetic signs.

### Gestures as *Visuo-Kinetic Signs* in Multimodal Contextures of Communicative Action

Co-speech gestures here are understood as discourse-embedded, kinetic action that is performed with the head, hands, arms, torso, or entire body and has some communicative function(s) (e.g., Müller, 1998; Kendon, 2004; Calbris, 2011). Partly in reference to Jakobson (1972, p. 474) notion of “motor signs,” the term *visuo-kinetic signs* is introduced here to encapsulate the fact that gestures are part of, or emerge from, the human body with its inherent morphology, motion range, motor routines, and multiple senses with which we perceive and understand the world around us. Gestures genuinely preserve and (re-)enact some of their kinetic, sensorimotor, tactile, and interpersonal origins (e.g., Mittelberg, 2010, 2018; Müller, 2017). While gestures usually need concurrent speech to specify their local meaning, they often *do* something in their own right and in their own specific, experientially motivated ways (e.g., Mittelberg and Joue, 2017; Müller, 2017; Wehling, 2017).

While gestures are part of the visual – and thus visible and observable – facets that make up contextualized language use in interaction, the Kendon’s (2004) idea of *visual action as utterance* duly emphasizes the fact that gestures are more than just visual. Gestures are communicative *bodily actions* that are instantaneously *performed* by human beings and dynamically *evolve* in time and space (e.g., Müller, 1998; Sweetser, 2007; Goodwin, 2011; Streeck et al., 2011). One important factor in gesture interpretation and analysis, however, resides in the fact that the ‘semiotic material’ we are looking at consists not only of observable physical components – such as body posture, body motion, finger configurations, as well as the position and movements of gesturing hands – but also of immaterial, yet signifying, components such as evanescent movement traces created in the air or imaginary surfaces, objects, or points in space (e.g., Mittelberg, 2010; Hassemer, 2015). Speakers’ hands often pretend to hold or otherwise manipulate virtual objects and/or tools – the typing gesture necessarily implies an imagined keyboard – or to interact with imaginary interlocutors. Consequently, to do justice to the noted specific semiotic nature of gestures, the present account of gestures as visuo-kinetic signs also includes elements and dimensions of multimodally achieved sign processes that are not visual, and hence rather invisible, but still contribute to a gesture’s kinesthetic feel, meaning, and pragmatic function(s) (e.g., Mittelberg, 2006, 2013). As will be shown below, metonymy enables us to account for the virtual elements thus implied, or created on-the-fly, which may be inferred from their dynamically evolving multimodal *semiotic contextures* (Jakobson, 1956; see also Müller, 1998; Streeck, 2009; Goodwin, 2011). By illuminating the pragmatic workings of metonymy in visuo-kinetic signs, this paper seeks to provide additional insights into the nature of both gesture and metonymy.

## Toward a Frame-Based Account of Embodied Metonymy in Gesture

Metonymy belongs – together with metaphor, synecdoche, and irony – to the four master tropes (Burke, 1941). Jakobson (1956) was one of the first to advocate a balanced theory of metaphor and metonymy as two universal principles of association and signification that are prominent in language, thought, discourse, literature, and the visual arts (e.g., Waugh and Monville-Burston, 1990). Subsequently, experientialist views on language and the embodied mind attributed a preeminent role to metaphor (e.g., Lakoff and Johnson, 1980; Johnson, 1987; Lakoff, 1987; Sweetser, 1990). The ground-laying idea was that the human conceptual system, language, language change, and language use, encompassing all types of discourse, are structured and function metaphorically to an extent that had previously been underestimated.

With little delay, metonymy has become recognized as an equally important figure of thought and language (e.g., Gibbs, 1994, 1999; Panther and Radden, 1999; Barcelona, 2000a,b, 2003; Dirven and Pörings, 2002; Fauconnier and Turner, 2002; Panther and Thornburg, 2003). In recent years, cross-linguistic research has clearly confirmed that metonymy plays a constitutive role in conceptual, semantic, and grammatical structuring, as well as in discourse processes, including, for example, indirect reference, speech acts, and pragmatic inferencing (e.g., Barcelona, 2009; Panther et al., 2009; Benczes et al., 2011; Kövecses, 2013; Littlemore, 2015). A crucial tenet of the present proposal on how gesturally engendered sign processes involve ‘metonymy in the making’ is that “metonymy is a central organizing principle of pragmatics, the contextual use and interpretation of meaning” (Dancygier and Sweetser, 2014, p. 162; see also contributions in Hampe, 2017).

Furthermore, there is a growing body of work on metonymy in various modalities, media, and art forms, ranging from mnemonic devices, painting, material culture to advertisement and film (e.g., Jakobson and Pomorska, 1983; Mittelberg, 2002, 2006; Forceville, 2007; Forceville and Urios-Aparisi, 2009; Littlemore, 2015). Regardless of the modality or medium in which metonymy materializes, it may create single meaningful sparks in our minds or set into motion complex associative chains and networks (e.g., Benczes et al., 2011). Metonymy thus may propel diverse processes of reasoning, imagination, and discourse construction (e.g., Coulson, 2001; Fauconnier and Turner, 2002; Dancygier and Sweetser, 2014), both within one modality and across modalities.

Due to limits of space, this paper cannot provide a comprehensive overview of all the different kinds, functions, and manifestations of metonymy described in the literature. Rather, I will draw on the approaches to metonymy that seem particularly apt to account for the structuring and meaning-making processes at work in bodily signs that partake in multimodal interaction. I will thus try to show why, in the case of manual gestures and other visuo-kinetic signs, it makes sense to shift the focus from strongly cognitively oriented accounts to truly embodied, or body-based, understandings of metonymy. To this effect, the exposition below will provide further gestural evidence for the claim that metonymy is more directly experientially motivated than metaphoric processes with which they tend to interact (building on Mittelberg, 2006, 2013, 2017a; Mittelberg and Waugh, 2009, 2014; see also, e.g., Barcelona, 2000b; Kövecses, 2013; Dancygier and Sweetser, 2014; Ruiz de Mendoza Ibanez, 2017).^3^ Advocating a frame-based account of metonymy in gesture, the ensuing sections aim to show how various metonymic principles function as fundamental construal mechanisms that drive pragmatically grounded processes of embodied schematization in co-speech gestures.

### Experiential and Functional Domains

According to domain-based accounts, metonymic mappings occur within the same cognitive or experiential domain, or within the same *idealized cognitive model* (i.e., ICM, Lakoff, 1987; Panther and Radden, 1999: 19ff.). Barcelona (2003, p. 83) provides the following definition with a functional emphasis: “(a) metonymy is a mapping of a cognitive domain, the source, onto another domain, the target. Target and source are in the same functional domain and are linked by a pragmatic function, so that the target is mentally activated.” Let us consider the by now classic example of a metonymic linguistic expression given in (1) (Lakoff and Johnson, 1980, p. 35; see also Dancygier and Sweetser, 2014, p. 5):

(1) The ham sandwich is waiting for his check.
(2) Table 5 urgently needs to pay.

Here, “the ham sandwich” does not refer to a food item but, indirectly, to the restaurant client who ordered it. The dish previously served by a member of the service personnel, and probably already consumed by the client, thus stands for the latter, based on contextual, pragmatic links binding these elements within one and the same functional domain (e.g., Fauconnier and Turner, 2002). Another common way to refer to restaurant clients is by the number of the table they are sitting at, such as in (2). Here, a different element in this particular experiential domain or pragmatic context is highlighted, namely the table as a physical location inside the restaurant. In both expressions, the client is the metonymic target domain. The kind of domain that is chosen to be the metonymic source domain depends on the pragmatic forces and customs at work in a particular context of use. The factors that determine the choice of source domain in (1) and (2) include the interpersonally built-up common ground and the professional practices of the service personnel, who are used to communicating about this kind of frequently occurring situation.

As is well known, metaphor, by contrast, involves a mapping between two different experiential domains, as expressed by, for instance, the conceptual metaphor UNDERSTANDING IS GRASPING (e.g., Lakoff and Johnson, 1980). This cross-domain mapping gives rise to metaphoric linguistic expressions, such as in (3), where the abstract target domain of understanding is conceptualized in terms of the physical source domain of manually seizing an object. Alternatively, the same target domain may be structured by another bodily source domain, that is, visual perception as a way of comprehending something (UNDERSTANDING IS SEEING, ibid.), such as in (4).

(3) Paula grasped the new idea right away.
(4) *Paula instantly saw what I meant*.

In gesture, body-centered and action-based source domains may intuitively activate pragmatic links to metonymic targets with which they are connected through repeated instances of similar physical experience (e.g., Mittelberg and Joue, 2017). Certain manual actions may evoke the objects or tools that are routinely handled when they are actually performed. For instance, to ask for more bread in a restaurant, one may first raise a hand to catch the waiter’s attention and then point with that hand at the empty bread basket one is holding up with the other hand. The waiter will readily understand this gestural request based on the gesture and the empty bread basket, which here functions as the source domain pointing to the desired target: additional bread. Put differently, the CONTAINER stands metonymically for the wanted CONTENT; arriving at the latter involves following a contextually shaped, inferential pathway (e.g., Barcelona, 2003, 2009; Panther and Thornburg, 2003). Bread basket and bread belong to the same mundane experiential domain not only in people’s homes, but also in a restaurant context, where it is common practice to serve bread in baskets and also to provide refills.

If one performs this gestural request without concurrent speech, the basket metonymically evokes the idea of bread on visual and experiential grounds. However, it is likely that the person wanting more bread actually also verbally asks the waiter for it, as in (5) or (6), once the latter has arrived at the table. In both linguistic examples, there is no mention of the basket, only of the (non-existent) bread. Furthermore, (6) functions as an indirect speech act, that is, an assertion indirectly functions as a request in this case. Qualifying as a *speech act metonymy*, it may be understood “as a *scenario* having metonymic structure” (Panther and Thornburg, 2003, p. 128).

(5) Could we have more bread please?
(6) We are out of bread.

Building on Langacker (1987), Croft (1993) extended the single-domain approach to a domain matrix, which involves shifts in foregrounding from one domain to another domain in the same matrix. Applying this idea to the bread-request scenario, we can assume that first the empty basket is foregrounded due to its perceptional prominence with respect to the bread that formerly existed in it; then, the metonymic process causes a shift in foregrounding onto the metonymic target, that is, the indirectly referenced bread that the client would like the waiter to fetch.

When it comes to gesture and multimodal interaction, the focus is naturally on the communicating human body and thus especially on those experiential domains that are indexically anchored in the material and social contexts of people engaged in some sort of physical action or in communicative exchange (Mittelberg, 2017a). For example, a participant in a study on transitive action gestures (Grandhi et al., 2011) gives the following verbal instruction regarding how to slice an apple into pieces:

(7) *You need to slice the apple by holding it down and cutting it there*.

**FIGURE 1:**
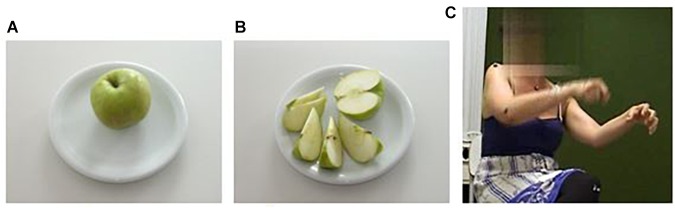
Photos **(A,B)** show a before-and-after scenario presented to participants who were asked to tell interviewers how to get from **(A)** to **(B)**. The video still **(C)** shows how gesture draws on the experiential domain of cutting an apple into slices; adapted from Grandhi et al. (2011). Written informed consent was obtained from the depicted individuals for the publication of this image.

Here no actual (i.e., visible) physical object is involved in the gestural portrayal. Pretending to be holding a virtual knife in her dominant right hand, she pantomimes how she would cut a virtual apple that she is seemingly holding down with her left hand (Figure 1, adapted from Grandhi et al., 2011).^4^

Indeed, slicing an apple into pieces necessitates a particular action (cutting), an object (apple), and a tool (knife). All three elements thus belong to the same experiential domain or scenario. Whereas what the participant says in (7) draws attention to the cutting action and the object, but not to the tool, the latter can be easily inferred from the action context. Again, the actions and objects implied belong to an everyday domain of experience. Moreover, this example involves a CAUSE-EFFECT metonymy, for we can imagine the apple first in its entirety and then the slices resulting from the cutting action (as shown in Figure 1; see also Mittelberg and Waugh, 2014).

### Semantic Frames and Familiar Scenes of Experience

A large part of what has been described above based on cognitive, experiential, or functional domains can also be understood in frame-semantic terms (see also, e.g., Panther and Radden, 1999, p. 9; Kövecses, 2013; Dancygier and Sweetser, 2014). According to Fillmore (1982, p. 111), the term *frame* covers “any system of concepts related in such a way that to understand any of them you have to understand the whole structure in which it fits.” Frames can thus be understood as metonymically structured wholes in which one of its parts may evoke another correlated part or the frame as a whole (e.g., Mittelberg and Joue, 2017).

While the semantic structures in question are situated at relatively high levels of abstractness, Fillmore (1975, p. 127) emphasizes how frames are experientially grounded in familiar *scenes* which underpin the acquisition of word meanings and the gradual differentiation of whole scenarios into their constitutive parts. Scenes “include not only visual scenes but familiar kinds of interpersonal transactions, standard scenarios, familiar layouts, institutional structures, enactive experiences, body image; and, in general, any kind of coherent segment, large or small, of human beliefs, actions, experiences, or imaginings” (Fillmore, 1977, p. 63). Since human behavior and gestures are intrinsic to such scenes and are also shaped by them, it seems fitting to exploit the notions of both frames and scenes to explicate gestural communication (e.g., Sweetser, 2012; Mittelberg and Waugh, 2014). As proposed in earlier stages of the present account (e.g., Mittelberg, 2017a,c; Mittelberg and Joue, 2017), gestures that recruit frame structures tend to metonymically pick out essential elements and salient qualities of scenes, that is, the motivating context of frames. This especially pertains to situated factors of real-world, enactive experiences that can be recruited for both literal and metaphorical construal and thus also involve *primary scenes* and *primary metaphor* (Grady, 1997; see also section “Metonymy Underpins Schematic Gestural Patterns and Fully Codified Visuo-Kinetic Signs”). The ways in which embodied metonymy plays a central role in frame-based processes that drive multimodal discourse pragmatics is discussed in the next section.

#### Frames and Frame Metonymy in Co-speech Gestures

Dancygier and Sweetser (2014: 102ff.) point out that, compared to domains, the structural organization of frames allows for a more fine-grained and systematic account of correlations not only within a frame (thus giving rise to *frame metonymy*), but also between two frames that are partially mapped onto each other (thus giving rise to metaphor). They provide the following general definition of metonymy: “the use of some entity A to stand for another entity B with which A is *correlated*” (ibid.: 134, italics in the original). Frame metonymy refers “to all usages where one reference to an element of a frame is used to refer to either the frame as a whole or to other associated elements of the frame” (ibid.: 135), for example, where ‘the Crown’ refers to the British monarchy. *Part-whole* frame metonymy includes what is generally understood by *synecdoche*, for example, where ‘field hands’ stands for people who work outdoors on a farm.

An often-cited example is the RESTAURANT DINING frame (or *script*, see Schank and Abelson, 1977); it implies elaborated scenarios involving certain culturally shaped sets of elements, roles, behaviors, and sequences of events (Fillmore, 1982). Seen from this perspective, Examples (1), (2), (5), and (6) discussed in Section “Experiential and Functional Domains” involve items that are integral to this frame structure: the guest who ordered the ham sandwich, the sandwich itself, Table 5, the bread basket, ordering more bread, and asking for the check. We are able to place and relate all these items within a structured, dynamic fabric of correlations that allows us to quickly understand acts of indirect reference and other metonymic operations occurring within it. Such larger frame structures, or scripts, are supposed to be active in the background processing of cognition and behavior (e.g., Coulson, 2001), in the sense that one becomes aware of them if an element is omitted or occurs sequentially out of place; for example, if someone asks for the bill before having consumed the dish that s/he ordered.

In processes of frame-based language use, reasoning, and discourse understanding, networks of metonymic relations inherent to specific frames thus become activated and operationalized (e.g., Coulson, 2001; Dancygier and Sweetser, 2014). Thereby, each frame structure provides various springboards for metonymic associations as well as entry points and conceptual bridges for intersubjective meaning construction in conversational exchanges or collaborative story telling. In ongoing interaction, speakers may use linguistic, gestural, or eye-gaze cues to frame a given scene in positive, critical, doubtful, or humorous terms, from a scene-internal or a scene-external viewpoint, or by adopting several viewpoints simultaneously (e.g., McNeill, 1992; Dudis, 2004; Sweetser, 2012; see Mittelberg, 2017b on the interplay of viewpoint, indexicality, and metonymy in gesture). Alluding to a particular discourse-relevant frame element may automatically trigger connections of different scope and varying complexity, for example, to directly correlated elements, the frame as a whole, or metaphoric associations.

With regard to the typing gesture described in the introductory section, a decisive detail lies in the fact that understanding the message involves a *cross-modally* instantiated, frame-internal metonymic process. So, again, if I imitate typing with both hands while saying to a friend:

(8) I’ll be working the entire weekend on my paper.

the pantomimed action of typing not only gets profiled against the ground of the imaginary keyboard, thus evoking the TYPING and WRITING frames, but also against the backdrop of larger frame structures, such as WRITING AN ACADEMIC PAPER or PUBLISHING. Note that in the verbal part of the utterance, the verb does not refer to the gesturally simulated typing action, but to the more general WORK frame. Hence, a cross-modal metonymic process takes place whereby the gesture specifies the verbally communicated information ‘I’ll be working’ as ‘typing’ or ‘writing’ a manuscript. For the interlocutors, this visuo-kinetic sign (including the imaginary keyboard) may instantly serve as a dynamically created *material anchor* (Hutchins, 2005) for joint attention and thus evoke aspects of their shared experience of such situations. In this way, webs of associations may branch out from such a mutual gestural anchor: In their respective embodied minds, this may facilitate associations that are not only directly grounded in physical experience, such as manipulating a keyboard or touchpad, but also bring to mind less tangible associations, such as subsequent phases of the work process, the potential structure and content of the paper, a previously co-authored paper, as well the community’s reaction (see also Calbris, 2011: 10f.). Through activating the WEEKEND frame, they might also think of what one misses out on while working the entire weekend. For both the speaker and the interlocutor(s), frame-metonymic associations are thus also likely to solicit subjective and intersubjective dimensions connected with certain mental or emotional states, such as being focused, anxious, or happily working away (see also Mittelberg and Waugh, 2014).

Regarding linguistic expressions, Dancygier and Sweetser (2014, p. 108) further emphasize that a certain degree of salience is needed to clearly associate a term with a frame in the sense of Langacker’s (1987) notion of *active zone* as the profiled part of a whole.^5^ For a body-based and action-based view of metonymic processes (Mittelberg, 2017a), it is particularly relevant that the human body forms a metonymically structured whole in and of itself. Certain parts of it, for example, the head or hands, may become prominent in a meaning-making process such as in the verbal example of ‘field hands’ mentioned above. In this kind of part-for-whole frame metonymy “the part centrally or directly involved in an activity stands for the whole. The hand, for example, is the part of the arm used for holding, touching, etc.; hence it is the active zone of the arm for many purposes” (Dancygier and Sweetser, 2014, p. 144).

Visuo-kinetic signs performed by heads, shoulders, and/or hands may also function as the signifying, active zone of the gesturer’s body that ‘stands out’ within dynamic multimodal contextures and may thus become meaningful. Furthermore, these signs may metonymically stand for the entire person making the gesture – or for the belief system behind a certain stance s/he is expressing toward what is being said (see also Calbris, 1990 on *body segments*).^6^

#### Gestural Frame Evocation at Varying Levels of Groundedness and Complexity

Building on Fillmore’s (1977, 1982) notion of semantic frames, Mittelberg (2017a) has recently presented a frame-based account of gesture pragmatics. It proposes different kinds of embodied frame structures that are situated at varying levels of groundedness, schematicity, and complexity, a synopsis of which will be presented here.

First, *basic physical action frames* and *basic object frames* are understood as being directly grounded in physical experience and basic scenes (Mittelberg, 2017a: 215ff.). These strongly embodied frames mainly encompass *prototypical events* (Slobin, 1985) such as pushing, pulling, and teasing apart; *mimetic schemas* (Zlatev, 2014) such as jump, kick, grasp, and hit; *basic-level actions* (Lakoff, 1987) such as eating, running, and walking; as well as any other intransitive, transitive or ditransitive actions that may be simulated via gestures and whole-body enactments (e.g., Hostetter and Alibali, 2008; Bressem and Müller, 2014; Müller, 2017). In addition, basic physical action frames may intertwine with basic object frames to account for the physical entities that the former, together with their affordances, typically imply (as in Figure 1, 4; see also Grady, 1997 on primary scenes).^7^ Basic object frames also get evoked in multimodal descriptions of physical entities or spaces.

Second, more *complex frame structures* comprise frames that are internally more differentiated, more detached from the motivating contexts of experience and hence situated at higher levels of abstractness (Mittelberg, 2017a: 220ff.). Presupposing the “development of a complex frame out of correlated simpler frames” (Dancygier and Sweetser, 2014, p. 138), we will first consider frame structures that are composed of several connected basic action or object frames and hence exhibit an intermediate level of groundedness. The RESTAURANT DINING script (see section “Frames and Frame Metonymy in Co-speech Gestures”), for example, consists of such a culturally shaped ordered set of basic actions and their implied objects and/or interacting persons that are fairly well grounded and may thus function as experiential anchors: being seated, looking at the menu, signaling to the waiter, eating, paying, etc. Each of the sequenced actions and behaviors involve physical activities and can thus be easily enacted through postures, gestures, and facial mimics, and hence evoke other, correlated items or the overarching frame as a whole.

*Highly abstract complex frame structures* are understood as being a lot more detached from motivating contexts than the frames discussed so far. They involve cognitive and semiotic structures and activities that people rely on when producing or describing phenomena at a meta-level, for instance, linguistic structures, genre-dependent narrative and conversational patterns, plots of novels, films, or animated cartoons, mental maps, as well as knowledge systems and schematic conceptual structures such as theories or category systems (Mittelberg, 2017a: 223f.). In gesture, such larger architectures may be overtly represented and thus become visible, albeit minimally and fleetingly, via virtual time lines traced in the air (e.g., Calbris, 2011) or other diagrammatic configurations of points placed in gesture space that highlight how individual words, items, places, events, concepts, or more general discourse contents relate to one another temporally, spatially, or logically (e.g., Kendon, 2004; Mittelberg, 2008; Enfield, 2009; Bressem, 2014). Beat gestures (McNeill, 1992) are also a means to accentuate particularly relevant parts of an utterance, thus making them metonymically stand out from the speech chain as a whole.

**FIGURE 2:**
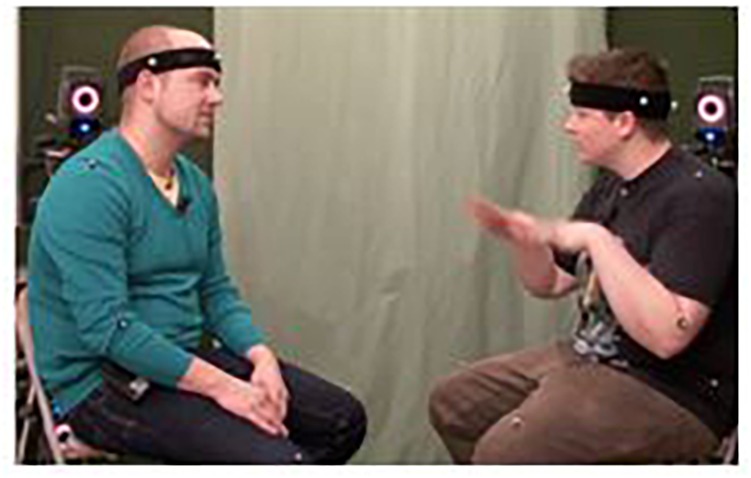
Speaker evoking the SWIMMING action frame in a reduced and partial metonymic fashion. Written informed consent was obtained from the depicted individuals for the publication of this image.

Let us now look at how basic and more complex frames may interact in organizing a thematic unit of multimodal discourse. Example (9) is taken from a description of a past vacation scene produced in the context of a travel-planning task. Suggesting Hungary as a possible destination on a joint trip through Europe, the person on the right in Figure 2 is telling her conversational partner in German that on a previous visit to Budapest the weather was very nice. By mentioning that it was beautiful outside (‘it… was really nice’), the speaker verbally evokes the WEATHER frame in an indirect fashion. She then profiles a specific sub-frame against the backdrop of the larger, general WEATHER frame by uttering the compound noun ‘*Schwimm*wetter’ (*swimming* weather).^8^

(9) es (…) war richtig schön und so [Schwimmwetter]
  (it (…) was really nice and like [swimming weather])

The simultaneously produced visuo-kinetic sign shown in Figure 2, consisting of simulated swimming movements performed by the speaker’s hands and arms, renders this specification salient. This gesture can be said to activate the *basic physical action frame* SWIMMING. Not all the body parts usually involved in swimming participate in this partial, stylized iconic enactment of a learned motor routine. With reference to Hostetter and Alibali’s (2008) *gestures-as-simulated-action* (GSA) framework, this gestural action exemplifies how “gestures emerge from the perceptual and motor simulations that underlie embodied language and mental imagery” (Hostetter and Alibali, 2008, p. 502). Under the present view, this is another example of how communicative gestures may metonymically evoke the physical actions they are imitating iconically by only minimally enacting the onset or some essential characteristics of a full-blown action routine in a rather schematic fashion.

**FIGURE 3:**
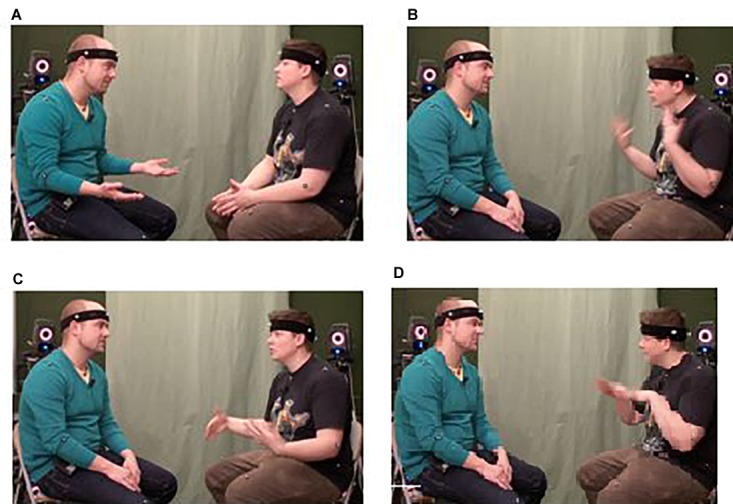
Multimodal evocation of larger frame structures: **(A)** The linguistic question evokes the WINTER frame, while the gesture pragmatically evokes the QUESTION (speech act) frame; the linguistic reply evokes the WARM WEATHER frame characterized by a series of gestures, each of which details a different basic object/action frame: **(B)** T-SHIRT; **(C)** SHORTS, and **(D)** SWIMMING. Written informed consent was obtained from the depicted individuals for the publication of this image.

Looking at the immediate discourse context of this bimodal performance reveals that it belongs to a vivid description, provided in (10), in which several gestures portray additional aspects that belong to what seems a general, yet culture-dependent, understanding of WARM WEATHER. Enquiring about the weather conditions on this past trip, the participant on the left actually first evokes the WINTER frame: He asks whether there was snow and simultaneously makes a bimanual Palm-Up Open Hand gesture (PUOH, Müller, 2004), shown in Figure 3A. This pragmatic gesture functions here as a visuo-kinetic question marker, or an interactive *seeking* gesture (Bavelas et al., 1995), that is soliciting an answer from his interlocutor in an ‘empty-handed’ manner (see also Kendon, 2004; Streeck, 2009; Bressem and Müller, 2014). His interlocutor then replies that it was actually rather nice and warm.

(10) P_Left_:   lag Schnee?                     [was there snow?]
          P_right_:  nein (–) es war schon       no (–) it was actually
                      warm                                warm
                      also so                              well like [t-shirt
                      [T-Shirt-Wetter] und        weather] and
                      [Shorts-Wetter] (…)        [shorts weather] (…)
                      war richtig schön             was really nice
                      und so                              and like [swimming
                      [Schwimmwetter].           weather]

When explaining that it was “t-shirt weather” the speaker on the right rotates both hands at approximately shoulder height with the palms facing toward the t-shirt she is wearing (Figure 3B). This gesture may be interpreted as pointing to the short sleeves of her t-shirt. Considered as a *cyclic gesture* (Ladewig, 2011), it may also evoke the idea of continuously feeling hot or of sensing hot air surrounding the body. The speaker then accompanies her verbal utterance “shorts weather” with another bimanual gesture: With the palms facing the torso, the outer edges of the hands indicate the location on her thighs where shorts typically end (Figure 3C). Only then does she multimodally activate the SWIMMING frame as described earlier (Figure 2, 3D). These individual, metonymically linked frame elements jointly draw on the WARM WEATHER frame as a whole. In this way, the semantic structures evoked in Example (10) involve several, metonymically correlated frame elements and are thus more complex than the individual basic physical action frame (SWIMMING) and the basic physical object frames (T-SHIRT and SHORTS) which constitute them. Larger frames at this intermediate level of groundedness are still rooted in habitual, mundane physical and social activities and thus may draw on various “scenes basic to human experience” (Goldberg, 1995, p. 5).^9^

Indeed, scenes have been found to be particularly relevant with respect to how interlocutors construe and follow processes of online meaning construction. According to Fillmore (1977, p. 1226), “in most natural conversations, the participants have, already ‘activated,’ a number of shared, presupposed, scenes that we can speak of as being in their consciousness as they speak.” This supports the idea that scenes partake in the dynamic contexts that shape multimodal processes of conceptualization during both the production and interpretation of co-speech gestures. A frame-based understanding of multimodal discourse pragmatics has the advantage of including larger semantic networks that go beyond local reference or individual simulated actions, thus leading into discourse-driven processes of more complex meaning construction.

Although the different kinds of frame structures discussed so far only pertain to concrete actions and objects, they may, in principle, also underpin metaphoric construal in gesture (e.g., Dancygier and Sweetser, 2014; Mittelberg and Joue, 2017). This line of inquiry also leads into related linguistic issues such as how embodied scenes and frame metonymy may factor into related syntactic frames, grammaticalization in gesture, as well as multimodally instantiated constructions (e.g., Mittelberg, 2017c; Zima and Bergs, 2017; see section “Metonymy Underpins Schematic Gestural Patterns and Fully Codified Visuo-Kinetic Signs”).

### Reference and Pragmatic Inferencing in Gesture

Exploring how metonymy motivates gestural practices of frame evocation necessarily raises questions concerning reference and inference. While these complex issues cannot be resolved here, let us pursue the idea that many gestures tend to *evoke frames* and *enact* or *simulate* physical actions rather than *represent* or *refer to* things or actions in the real world (e.g., Merleau-Ponty, 1962; McNeill, 2005; Mittelberg, 2018). Unlike spoken and signed languages, most spontaneous gestures do not rely on fully coded form-meaning pairings on the basis of which referential processes typically function. Rather, *habituated inferences* based on habitual actions as well as habits of gesture production and interpretation seem to play a central role in how gestures signify. Here, a parallel may be drawn with how metonymy plays a role in catalyzing inferential and referential interactions in language, as Barcelona points out:

Metonymy has this inferential role because of its ability to mentally activate the implicit pre-existing connection of a certain element of knowledge or experience to another. The referential function of metonymies is thus a useful (hence extremely frequent) consequence of their inference-guiding role since what we do when we understand a referential metonymy is to infer the referential intentions of others (Nerlich and Clarke, 2001; Barcelona, 2009, p. 369).

Metonymic inferences in co-speech gestures may occur within the gestural modality or cross-modally, that is, triggered by a linguistic cue (e.g., Mittelberg, 2017a). Experientially entrenched pragmatic inferences (Dancygier and Sweetser, 2014, p. 144) are indeed key to mentally simulating and understanding the communicative intentions of the gestural actions performed by others. As we saw earlier, Figure 1 demonstrates a habitual metonymic correlation between gesturing hands and the cutting action they are simulating: the apple is seemingly being held down by one hand, while the seemingly held knife in the other is not referred to in the speech chain, but implied in the action. Performing an “inference-guiding role,” the gesture here can be said to activate an “implicit pre-existing connection of a certain element (…) of experience to another” (Barcelona, 2009, p. 369). Gesturally triggered metonymic pathways of this nature may be seen as *natural inference schemata* (Panther and Thornburg, 2003, p. 8) or *vital relations* (Fauconnier and Turner, 2002: 93ff.): they intuitively draw on people’s embodied, situated ways of functioning not only in the physical world, but also in imaginary and/or abstract worlds (e.g., Sweetser, 2007, 2012).

For example, arriving at the contextualized meaning of the quick gestural indications in Figure 3 relies on several cross-modal processes of pragmatic inferencing. Understanding these gestural portrayals as illustrating the WARM-WEATHER frame requires integrating the verbal utterance in (10) with information that is made visually salient. Apart from the iconic swimming gesture, the other frame elements mentioned in speech, such as the t-shirt and shorts, are evoked in a rather approximate way. In this multimodal portrayal, we can identify the following inferential pathways: two lead from the gesturing hands to the respective body parts and indicated items of clothing. Through the indexicality inherent to these gestures, what they allude to briefly constitutes a signifying, active zone (Langacker, 1987) that is profiled and thus perceptually foregrounded in this instance of multimodal meaning elaboration (see section “Experiential and Functional Domains”). Together these pathways lead more globally into the WARM-WEATHER frame, in the context of which these specific garments are commonly worn in combination. In these cases, but also more generally, the concurrent speech content is needed to disambiguate, via inferential processes, especially those gestures that only vaguely allude to something in the interlocutors’ environment or evolving discourse context.

So, although reference is one of metonymy’s chief functions, processes of pragmatic inferencing are often more crucially involved in assuring a gesture’s communicative function, at least from the perspective of the interpreter. Further gesture research is clearly needed to gain a fuller understanding of how speech and gesture interact in cross-modal processes of pragmatic inferencing including those that involve less accessible targets, for instance, through metaphoric construal (Mittelberg, 2006, 2017a).^10^ We will now look more closely at the junctures where such inferences tend to take place within visuo-kinetic signs.

### Contiguity Relations Operationalized in Co-speech Gestures

From a semiotic perspective, similarity (iconicity), contiguity (indexicality), and conventionality (symbols, habits) constitute the three fundamental semiotic relations that may be established between a material sign carrier and what it signifies; in any given sign process, they typically mix to varying degrees (Peirce, 1960). The present proposal emphasizes that motivation in gesture relies on both similarity and contiguity and that both modes usually also interact with various pressures of conventionalization (e.g., Mittelberg, 2006, 2013, 2014). According to Peirce (1960), contiguity encompasses different kinds of factual connections, notably physical impact, contact, and adjacency, as well as temporal and spatial proximity or distance. All of these may underpin indexical sign processes in which the material sign, for example, fingerprints left at a crime scene, points the interpreting mind toward the “object,” namely the person whose fingers caused traces of their impressions to adhere to surfaces through physical contact. Generally speaking, there are innumerable latent contiguity relations out there in the world, in our imagination, and in our embodied knowledge structures that may be operationalized when we are reasoning and communicating. This section will focus on contiguity relations that the speaker’s body forms with the physical or the imaginary world at her/his fingertips and that are intuitively drawn upon for multimodal meaning-making (cf. Table 1 for an overview of the Peircean and Jakobsonian semiotic concepts discussed in this section).

**Table 1:** Overview of semiotic foundations of metonymy in visuo-kinetic signs, drawing on Peircean semiotic theory and Jakobson’s view of metonymy as being derived from (outer and inner) contiguity and indexicality (as discussed in section “Contiguity Relations Operationalized in Co-speech Gestures”).

Semiotic relations (Peirce)	Semiotic modes (Peirce)	Metonymic principles grounded in contiguity and indexicality (Jakobson)
Similarity	Iconicity	**Inner *contiguity*** relations underpinning internal metonymy (e.g., partial, stylized iconic gesture standing for entire action involving more body parts and complex action routine)
Contiguity	Indexicality	**Outer *contiguity*** relations underpinning external metonymy (e.g., gesture for object involved in imitated action)
Conventionality habit	Symbolicity	**Habitual**/**conventionalized** metonymic operations (involving iconicity, indexicality, and symbolicity) • **Habitual** actions and pragmatic inferences operationalizing **inner/outer *contiguity*** relations (e.g., engendering gestural patterns) • **Coded** metonymic operations drawing on **inner/outer *contiguity*** relations (e.g., underpinning sign language lexemes)


Within cognitive linguistics, contiguity relations underpinning metonymic expressions are understood as either objectively given or cognitively construed (e.g., Panther and Radden, 1999; Dirven and Pörings, 2002). They are assumed to be contingent (Panther and Thornburg, 2003), that is to say, they may be canceled. These views are highly relevant to bodily semiotics and visuo-kinetic signs, for gesticulating hands typically do not manipulate real physical objects or surfaces; they only pretend to do so [as in simulating typing a paper, Example (8)]. In their prototype approach to conceptual contiguity and metonymy, Peirsman and Geeraerts (2006) posit the spatial and material domain as the prototypical core of contiguity. They present a continuum of strength of contact as the basis for spatial, temporal, as well as abstract domains (including events, actions, processes, and assemblies). For instance, in the spatial/material domain, the continuum extends from spatial (i) part/whole (e.g., head/person) to less prototypical cases, such as (ii) containment/container (e.g., milk/glass), (iii) location/located (e.g., house/inhabitants), and (iv) entity/adjacent entity (e.g., person/clothing). The first is equivalent to a part-whole-frame metonymy. Reflecting diminishing degrees of strength of contact, the second captures the relationship between a bread basket and bread (as discussed in “Metonymy Underpins Schematic Gestural Patterns and Fully Codified Visuo-Kinetic Signs”), the third captures the relationship between a table and a client sitting at a table [as in Example (2)], and the last captures the connection between the speaker’s legs in Example (10) and the shorts she refers to verbally. We will now consider a view of contiguity that makes comparable distinctions, but places emphasis differently.

Jakobson’s (1956) account of contiguity relations has proven to be particularly suitable to describe the functions that metonymy may assume in experientially motivated gestural signs (Mittelberg, 2006, 2010, 2013; Mittelberg and Waugh, 2009, 2014). In his writings on aphasic disorders, Jakobson (1956) showed just how deeply rooted the distinction between similarity (iconicity/metaphor) and contiguity (indexicality/metonymy) is. Furthermore, he differentiated contiguity relations in the physical world, for example, between a knife and a fork, and those which combine items in a semiotic contexture, for example, linguistic units jointly forming a syntagm or a discourse (Waugh and Monville-Burston, 1990; Dirven and Pörings, 2002; Hopper and Traugott, 2003). Of particular relevance to understanding how metonymy is operationalized in gesture is Jakobson’s distinction between *inner contiguity* and *outer contiguity*. The following visual scene serves to illustrate these different operations, which will be applied to gesture below:

One must – and this is most important – delimit and carefully consider the essential difference between the two aspects of contiguity: the exterior aspect (metonymy proper), and the interior aspect (synecdoche, which is close to metonymy). To show the hands of a shepherd in poetry or the cinema is not the same as showing his hut or his herd […]. The operation of synecdoche, with the part for the whole or the whole for the part, should be clearly distinguished from metonymic proximity. […] the difference between inner and outer contiguity […] marks the boundary between synecdoche and metonymy proper.

(Jakobson and Pomorska, 1983, p. 134).

#### Inner Contiguity: Parts, Phases, Contours, and Essential Qualities

Inner contiguity underlies part-whole relationships, that is, between a part and another part, a part and the whole, or the whole and the part (Jakobson and Pomorska, 1983). *Internal metonymy* operationalizes these kinds of contiguity relations inherent to a given gestalt. For instance, in *everyone lives under one roof*, ‘roof’ evokes the entire house of which it constitutes a physical fragment. Hence, internal metonymy entails that the inner structure of a body, entity, or action is broken down into its component parts, phases, or any other characteristic, and that one of them is taken to imply a connected component or the entire gestalt structure.

**FIGURE 4:**
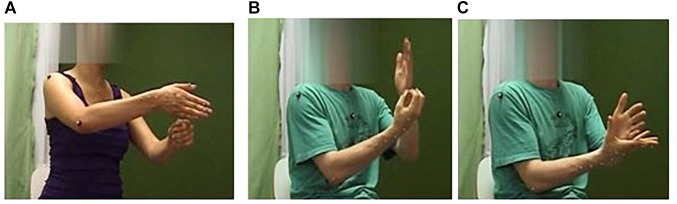
Gestural examples of internal metonymy: the hands turn into an apple and a knife to portray how to slice an apple: the outspread fingers of the right hand in **(C)** also evoke the slices resulting from the cutting action. Video still **(A)** corresponds to Example (11); video stills **(B,C)** correspond to Example (12) (adapted from Grandhi et al., 2011). Written informed consent was obtained from the depicted individuals for the publication of this image.

In manual gestures and whole-body enactments, internal metonymy establishes a predominantly iconic ground for signification (Peirce, 1960; Sonesson, 2007; Mittelberg, 2013, 2014). That is, it relies upon a metonymic rendition of what it signifies based on a perceived or construed similarity. Internal metonymy may thus motivate processes of profiling and highlighting prototypical, or locally salient, aspects of a given, existent or imagined, experience or gestalt. For instance, a gesturally enacted onset, path, or manner of motion may evoke, in an abstracted and idealized manner, the corresponding, fully articulated physical action (e.g., the swimming gesture in Figure 2) or motion event (e.g., McNeill, 1992). It is via metonymy that iconic gestures may also give salience to contours, shapes, spatial dimensions, and other relevant qualities of objects, spaces, and other kinds of physical structures (Mittelberg and Waugh, 2014). In the study on transitive action gestures mentioned in Section “Experiential and Functional Domains” (Grandhi et al., 2011), an alternative way of enacting the apple-slicing scenario (Figure 1) was to use the hands as if they were the apple and the knife, respectively, rather than pretending to handle them. Figure 4 shows two slightly different variants of this gestural technique, exemplifying the *representing* mode, according to Müller (1998, 2014).

(11) You need to slice the apple.
(12) You need to cut the apple.

In Figure 4 [Examples (11) and (12)], both of the participants’ hands exemplify the working of internal metonymy: The flat, vertically held hand looks and functions like the blade of a knife, that is, like the part of the kitchen tool would actually cut into an apple; the other, non-dominant hand forms a fist, thus resembling a round object, which, in this case, signifies an apple. Furthermore, the participant shown in Figure 4B,C opens up his hand, representing the apple, at the very moment when ‘the knife’ hits it, so that his fingers may be taken to iconically portray the apple slices resulting from the repeated cutting action in a schematic and partial fashion. In this visually effective instance of a gestural CAUSE-EFFECT metonymy, the semiotic affordances of the manual articulators are thus exploited to a great extent.

#### Outer Contiguity: In Touch With the Physical, Social, and Imagined World

Outer contiguity underlies metonymic expressions in which the profiled element is not part of, but externally contiguous and/or pragmatically related to the element that it enables an addressee to infer. *External metonymy* may draw on various kinds of outer contiguity relations and imply different degrees of metonymic proximity, such as contact, adjacency, impact, and cause/effect (Jakobson and Pomorska, 1983, p. 134). For instance, with respect to the metonymic source expressions “the ham sandwich” [Example (1)] and “Table 5” [Example (2)] referring to a restaurant client, the relevant contiguity relations hold between the client and the dish ordered earlier (temporal contiguity) and the table s/he is sitting at (spatial contiguity).

In gesture, contiguity holds between hands and the objects, tools, and surfaces with which speakers are (seemingly) in touch when communicating. Indexically anchoring the give and take of conversational exchanges in the actions of the human body, the material and social environment, or in imagined spaces (Sweetser, 2012; Mittelberg, 2017b), gestures readily (re-)establish and highlight such relations by instigating metonymic modes that operate at junctures of gesturing hands and contiguous persons or entities (as in the transitive action gesture in Figure 1).^11^

**FIGURE 5:**
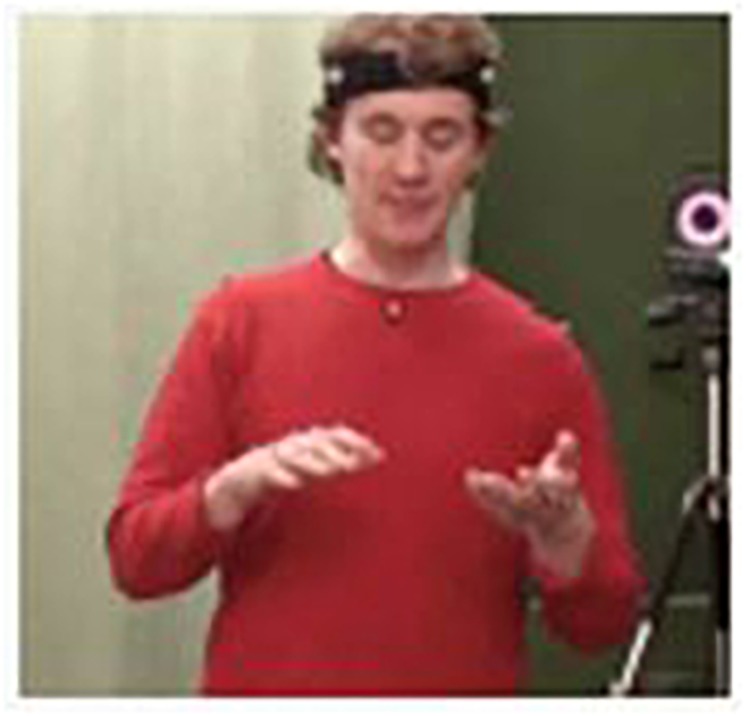
Gesture exemplifying an outer contiguity relationship between the left open flat hand and a virtual object seemingly placed on it. A muted index anchors a cross-modal process of pragmatic inferencing: the abstract discourse content, an analogy metaphorically construed as a tangible entity, can be inferred from the surface of the open hand via external metonymy (Example 13). Written informed consent was obtained from the depicted individuals for the publication of this image.

In Figure 5, for instance, the purpose of the gestural enactment is not to iconically imitate someone who is holding something. While this is, via internal metonymy, the perceivable starting point of the enacted meaning construction, it is the imaginary entity, externally contiguous to the PUOH seemingly supporting it, that the speaker is verbally drawing attention to. In Example (13), the architecture student is describing an analogy between the architectural design process and a musical episode. Due to the basic physical action frame of holding a physical object, it is easy to infer a generic object. The latter here stands for an abstract concept, an analogy, via an embodied metonymic inference mechanism based on immediate metonymic proximity of the open palm and the imagined object. It is through action-based, metaphorical reification that the analogy becomes a tangible and thus intersubjectively sharable element in the discourse context (Mittelberg, 2008; Mittelberg and Joue, 2017).

(13) Es gab ja die Analogie zur… zur Musik, also… oder…. oder auch zu ‘ner Interpretation
  (‘There was the analogy to… to music, so… or… or even to an interpretation’).

Under the present view, it is through a cross-modal process of pragmatic inferencing and a low degree of indexicality (Mittelberg, 2017a,c) that the PUOH is pointing to the existence and relevance of the verbally referenced analogy. Concurrently, the speaker’s right hand with the palm turned downward is oriented toward his left hand and thus creates an additional index leading to the analogy. This gesture thus heightens the relevance of an idea that seems to be physically graspable through the ongoing multimodal description.

These observations further support the idea that the source meaning, embodied in the form of a hand configuration and/or movement, remains present and perceptually salient in metonymic mappings, while the target meaning, that is, the discourse contents (such as the analogy in Figure 5) is cognitively prominent in the ongoing exposition (e.g., Panther and Thornburg, 2004, p. 95, 105; Mittelberg, 2006). We can also say that such object-oriented action gestures may trigger a frame-internal metonymic shift at outer contiguity junctions constituted by the hands and the virtual objects and tools they seem to be holding or manipulating (Mittelberg and Waugh, 2014). For example, the apple-slicing scenario in Figure 1 exemplifies the underlying metonymic mapping ACTION-FOR-OBJECT INVOLVED IN ACTION; in addition, the gesture in Figure 5 simultaneously relies on the relation PRESENTATION-FOR-PRESENTED (e.g., Panther and Radden, 1999; Panther and Thornburg, 2003; Barcelona, 2009; see also Mittelberg and Joue, 2017 on *gestural framing actions*). In both cases, what we actually see are the physical actions, but through following the linguistic cues in the unfolding discourse our attention shifts to the implied items and ideas.

A recent study combining behavioral and brain-imaging experiments (Joue et al., 2018) provides some initial neuroscientific evidence for processing differences that seem to broadly reflect the metonymic principles distinguished by Jakobson and Pomorska (1983) and discussed in this section. The study participants were shown video recordings of persons verbally describing and gesturally performing actions in which an object/tool in question was either represented by a finger/hand (internal metonymy or *body-part-as-object*) or the person was pretending to be holding or otherwise manipulating an object or tool (external metonymy or *pantomime*; see, e.g., Lausberg et al., 2003). Results suggest that metonymy may guide an interpreting mind to focus primarily on either locally relevant features (part-for-whole metonymy) or more globally relevant aspects (frame metonymy) of what is being communicated (Joue et al., 2018; see also Grandhi et al., 2011 on a user study showing clear preferences for external metonymy).

### Experiential, Metonymic Bases for Metaphoricity in Gesture

Metonymy and metaphor have been found to interact to varying degrees in language and other multimodal forms of communication (e.g., Jakobson, 1956; Goossens, 1990; Barcelona, 2000a; Radden, 2000; Mittelberg, 2002, 2008; Panther et al., 2009; Benczes et al., 2011; Kövecses, 2013; Littlemore, 2015; Hampe, 2017; Ruiz de Mendoza Ibanez, 2017). Investigating how indexical and iconic principles jointly guide the interpretation of predominantly metaphoric gestures, Mittelberg and Waugh (2009) suggest two distinct but intertwined semiotic processes in which metonymy leads into metaphor. For example, to reconstruct the meaning of the gesture evoking an analogy in Figure 5, we can first assume a process of metonymic inferencing as described in Sections “Reference and Pragmatic Inferencing in Gesture” and “Contiguity Relations Operationalized in Co-speech Gestures.” The metonymic source, namely the flat open hand involved in the source action (Mittelberg and Joue, 2017) of holding something, points to the adjacent metonymic target: the virtual object involved in the action. Second, the same imaginary object serves as the source of the metaphoric mapping whose target is the abstract notion of analogy referred to verbally (see also Taub, 2001 and Meir, 2010 on *double mappings* in sign language). Note that in this example of a gesturally enacted metaphor, the concurrent speech is non-figurative (see also Cienki and Müller, 2008; Mittelberg, 2008, 2014).

Furthermore, the gesture in Figure 5 is an instance of a gesturally expressed *primary metaphor* (Grady, 1997; Hampe, 2017), namely IDEAS ARE OBJECTS. It thereby evokes the basic physical action and object frame of handling objects (Mittelberg, 2017a), which involves a primary scene (Grady, 1997), a prototypical event (Slobin, 1985), and a scene basic to human experience (Goldberg, 1995; as discussed in Section “Gestural Frame Evocation at Varying Levels of Groundedness and Complexity”). It is hence central to the present perspective on multimodal metonymy that “(f)rame metonymy is closely tied to the kind of correlations which are involved in experientially based metaphors, in particular Primary Metaphors (…). It is precisely the development of a complex frame out of a correlated simpler frame which makes a primary scene so powerful” (Dancygier and Sweetser, 2014, p. 137). We can draw from these insights that metaphoricity in gesture needs to be analyzed in view of its experientially grounded, metonymic bases, which may be predominantly iconic or predominantly indexical (e.g., Mittelberg and Waugh, 2014). This also further supports the idea that metonymy is experientially more basic than metaphor. (Cf. Table 2 for an overview of the different approaches to metonymy discussed in this section).

**Table 2:** Overview of experiential and cognitive bases and the corresponding metonymic operations in accordance with cognitive linguistic approaches to metonymy.

Experiential and conceptual bases	METONYMY	Metaphor
**Domains** (Section “Experiential and Functional Domains”)	Metonymic operations profiling certain elements/qualities within the same • Experiential/functional domain or domain matrix • Idealized Cognitive Model (ICM) or cultural model	Crossmodal metonymy-metaphor interaction:
**Frames and scenes** (Section “Semantic Frames and Familiar Scenes of Experience”)	• Frame metonymy and part-for-whole frame metonymy • Metonymic organization of semantic/syntactic frames	Correlations between profiled parts across experiential domains/frames, e.g.,:
**Gestural frame evocation** (Section “Gestural Frame Evocation at Varying Levels of Groundedness and Complexity”)	Gestures metonymically profile salient elements of **scenes** and **frame structures** at varying levels of groundedness, schematicity, and complexity: • Basic object/action frames • intermediately complex frames • highly complex and abstract frame structures	Embodied metonymic bases of metaphoricity: (a) Gestural action evokes the object involved in the action via metonymy; (b) The same imagined tangible object signifies an abstract idea via metaphor.
**Reference/inference** (Section “Reference and Pragmatic Inferencing in Gesture”)	• Referential metonymy and indirect reference • Pragmatic inferencing guided by embodied habits of action and interpretation and/or linguistic cues	(see section “Experiential, Metonymic Bases for Metaphoricity in Gesture”)
**Contiguity** (Section “Contiguity Relations Operationalized in Co-speech Gestures”) (Table 1)	Metonymic correlations based on physical/conceptual contiguity. Contiguity relations get operationalized via predominantly iconic or indexical modes in gesture and/or linguistic cues: • *Inner contiguity* (internal metonymy; synecdoche), e.g., profiling quality or phase of an action • *Outer contiguity* (external metonymy), e.g., between hand and object involved in action	Interacting metonymic and metaphoric processes may propel • Pragmatic functions • Schematization • Grammaticalization (see section “Enacted Schematicity: Pragmatically Driven Patterns in Co-speech Gesture”)


*In addition, the table includes an application of semantic frames to gestures, devising varying levels of groundedness, schematicity, and complexity in gestural frame evocation, as well as aspects of metonymy-metaphor interaction in gesture.*

## Metonymy Underpins Schematic Gestural Patterns and Fully Coded Visuo-Kinetic Signs

Throughout the foregoing discussion, we have seen how metonymic modes may motivate various processes of experientially grounded abstraction and schematization with respect to particular gestures. We will now consider how metonymy may be said to also underpin the emergence of gestural patterns (in section “Enacted Schematicity: Pragmatically Driven Patterns in Co-speech Gesture”) as well as fully coded visuo-kinetic signs (in section “Metonymic Principles Operating in Signed Languages”).

Although gestures and signed languages largely share the same articulators and space as a medium of articulation, they also differ in the ways in which they are ‘visual’ and act as signs (e.g., Liddell, 2003; Wilcox, 2004c; Sweetser, 2009; Perniss et al., 2010; Kendon, 2014; Müller, 2017). In many discourse contexts, spontaneous gestures can afford to be quite allusive, idiosyncratically reduced semiotic gestalts, for they do not need to fulfill well-formedness conditions in the way that emblems and linguistic symbols in signed languages do. Gestures may in fact push metonymic form reduction and schematization to quite extreme degrees. This is partly because, most of the time, gestures do not carry the full load of meaning-making: The concurrent spoken utterance gives them a hand, so to speak, thus disambiguating potentially polysemous hand shapes and movements (e.g., Müller, 1998; Calbris, 2011).

A central point that this paper wishes to make is that using the umbrella term *visuo-kinetic signs* to encompass co-speech gestures and signed languages allows us to elucidate some commonalities regarding certain core principles of metonymically driven sign constitution and interpretation.

### Enacted Schematicity: Pragmatically Driven Patterns in Co-speech Gesture

A central goal in gesture research has been to identify patterns in gestural practices within and across individual speakers, languages, discourses, contexts, communities, and cultures (e.g., Kendon, 2004; McNeill, 2005; Streeck et al., 2011). Certain co-speech gestures have indeed been found to exhibit relatively high degrees of patterning and conventionality. Under the present view on regularities in gesture, *conventionality* strongly pertains to the Peirce (1960) notion of *habit*, rather than to imposed, *symbolic* codes in the narrow sense of the term (Mittelberg, 2006). Highly frequent and routinized gestures, particularly those that (also) fulfill pragmatic functions, indeed show an increased ‘visibility’ in multimodal interaction: for example, *gesture families* (e.g., Kendon, 2004); *recurrent gestures* such as the PUOH (Müller, 2004, 2017) or the *cyclic gesture* (Ladewig, 2011, 2014; see also Bressem, 2014; Bressem and Müller, 2014); and/or gestures enacting embodied image and force schemas (e.g., Mittelberg, 2008, 2018; Cienki, 2013; Wehling, 2017).^12^

Suggesting that scenes basic to human experience (Goldberg, 1995, p. 5) may underpin entrenched patterns in both language and gesture, it was argued in Section “Toward a Frame-Based Account of Embodied Metonymy in Gesture” (drawing on Mittelberg, 2017a and Mittelberg and Joue, 2017) that certain gestures tend to metonymically profile salient aspects of deeply embodied, routinized aspects of scenes, that is, the motivating context of semantic frames (Fillmore, 1977, 1982). The next logical steps of this rationale involve examining how metonymy conditions gradual, pragmatically motivated processes of grammaticalization (Hopper, 1998; Hopper and Traugott, 2003; Bybee, 2010) in gesture, how the resulting schematic gestures evoke correlated syntactic frames (e.g., Goldberg, 1995, 1998), and how they may partake in multimodally instantiated constructions (e.g., Mittelberg, 2017c; see also contributions in Zima and Bergs, 2017). A full account of these complex phenomena cannot be provided here, but we will see an example of how metonymy factors into gestural schematicity below.

Regarding the meaning of constructions in language, Barcelona (2009) ascribes a fundamental role to metonymy and pragmatic inferences (see also Panther et al., 2009). According to the present, admittedly preliminary, consideration of comparable processes in gesture, habituated physical actions and repeated similar acts of gesturing involve metonymy through propelling the establishment of not only individual, metonymically reduced gestures, but also more schematic gestural patterns, notably via discourse-driven routinization (e.g., Haiman, 1994; Hopper and Traugott, 2003) of certain physical actions. Such commonly used, more strongly conventionalized, visuo-kinetic signs should evidence the metonymic processes discussed in this paper to high degrees. Gestures displaying this increased level of *embodied schematicity* are likely to combine referential (including metaphoric) and pragmatic functions, and their interpretation can be expected to rely on entrenched processes of pragmatic inferencing (such as the ones described in section “Reference and Pragmatic Inferencing in Gesture”).^13^

For instance, basic manual actions, such as holding or giving something to someone, have been shown to entail schematic scenes that underpin prototypical cases of transitive or ditransitive argument structure in language (Goldberg, 1995). In German, the full verb *geben* (give), a three-place predicate, underwent a process of grammaticalization engendering the existential construction *es gibt* ‘it gives’ (there is/are; Newman, 1998). In a recent study on multimodal instantiations of this impersonal construction (Mittelberg, 2017c), it has been argued that the manual action of giving also serves as *experiential substrate* in processes of embodied grammaticalization that result in gestural existential markers observed to co-occur with *es gibt*. These gestural markers tend to enact reduced and more schematic variants of the full action of giving. To illustrate this point, let us revisit the PUOH gesture in Figure 5 (discussed in section “Outer Contiguity: In Touch With the Physical, Social, and Imagined World”). In his left hand, the participant is seemingly holding the analogy he is talking about while using an *es gibt* construction to refer to it verbally (Example 13). The basic sense of the full verb *geben* (give) as well as the basic scene it evokes still resonate not only in this intransitive linguistic construction, but also in the gestural enactment that co-occurred with it. In essence, metonymic reduction can be said to motivate such frequently occurring, schematic communicative gestures out of fully fledged, object-oriented physical actions that originally involve object transfer. The act of giving is reduced to an act of unimanual holding that exhibits a decreased degree of transitivity and iconicity, thus evoking, for instance, a scene of existence, or presence, rather than a scene of object transfer.

These grammaticalized gestural markers of existence tend to stay rather close to the speaker’s body instead of reaching toward an (imagined) receiver. The hands are also more relaxed and reveal less effort than would be necessary to actually hold something. In addition, these visuo-kinetic existential markers tend to combine referential dimensions, afforded through metonymy, with modal or epistemic, that is, pragmatic functions (e.g., Sweetser, 1990). They also tend to express subjective and interactive dimensions of meaning (e.g., Hopper and Traugott, 2003), such as in Example 13, where the speaker points out something that seems obvious to him (for further details and examples see Mittelberg, 2017c; see also Bressem and Müller, 2014; Müller, 2017). Reduced degrees of iconicity and indexicality seem to push such commonly used gestures closer toward the juncture of *habit-driven, embodied grammaticalization* and gesture pragmatics. Further research is clearly needed to establish how these initial insights play out across speakers, languages, and discourse contexts.

The observations discussed here are akin to work on grammaticalization in signed languages, in the context of which gestures have been shown to serve as the *substrate* of certain lexical and/or grammatical signs (Janzen and Shaffer, 2002). Bearing this in mind, we will now turn to how metonymy operates in signed language.

### Metonymic Principles Operating in Signed Languages

Metonymy has been ascribed an important role in the construction of form and meaning in signed languages, for example, in ASL (e.g., Mandel, 1977; Taub, 2001; Liddell, 2003; Wilcox et al., 2003; Wilcox, 2004a,b), French Sign Language (LSF; e.g., Bouvet, 1997), German Sign Language (DGS; e.g., Kutscher and Lincke, 2012); and Israeli Sign Language (ISL; e.g., Meir, 2010; Meir and Cohen, 2018 fc.). For instance, investigating how iconicity and metaphor interact in ASL, Taub (2001) suggests a set of principles of sign constitution including *image selection, schematization*, and *encoding*. Metonymy particularly comes into play at the image selection stage: The ASL sign for ‘academic degree’, for instance, consists in showing the gestalt and length of a rolled-up diploma shaped like a cylinder. The sign portrays a tangible element that is pragmatically correlated with the target meaning within the same frame: “The degree itself is a non-physical title, rather than a physical object, and so a salient object is chosen for the purposes of creating an iconic sign” (Taub, 2001, p. 46).

**FIGURE 6:**
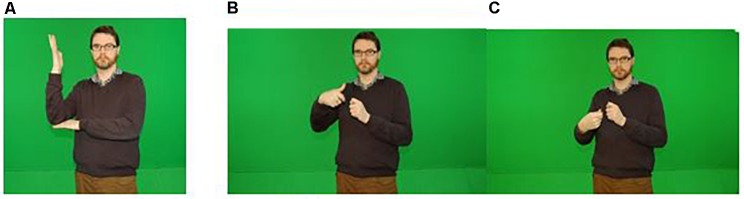
Photo **(A)** shows the DGS sign for ‘Baurrr’ (tree) exemplifying *internal metonymy:* photos **(B,C)** show the DGS sign for ’Banana,’ exemplifying *external metonymy.* Written informed consent was obtained from the depicted individuals for the publication of this image.

Let us now see how internal and external metonymy (as introduced in section “Contiguity Relations Operationalized in Co-speech Gestures”) are manifested in DGS. In Figure 6A, the lexical sign for ‘Baum’ (tree) exemplifies the workings of internal metonymy in the form of a bimanually achieved schematic icon that profiles the salient, structural parts of a tree, namely its trunk and branches, as well as the ground in which it is rooted. By contrast, the DGS sign for ‘Banane’ (banana) is a good example of how outer contiguity relations between the hands and a manipulated object are drawn upon to pragmatically infer what is signified (Figure 6B,C). While internal metonymy underpins the iconic hand shapes and movements as such, it is via external (or frame) metonymy that the hands’ shapes and actions evoke the implied (invisible) fruit. Although the peeling action is physically made salient, it is the object undergoing the action that is being referred to via this iconic visuo-kinetic lexeme. The latter may be said to evoke a basic action and object frame as discussed in section “Reference and Pragmatic Inferencing in Gesture” (Mittelberg, 2017a), involving the metonymic mapping ACTION-FOR-OBJECT INVOLVED IN ACTION (see section “Toward a Frame-Based Account of Embodied Metonymy in Gesture”; see also Wilcox et al., 2003).

## Concluding Remarks

The insights offered in the foregoing discussion provide further support for the idea that metonymy is a fundamental principle that operates across different modalities of experience, thought, and expression. The chief goal of this paper was to characterize and evidence the inherently metonymic nature of co-speech gestures. Combining cognitive linguistic and semiotic perspectives on how embodied metonymic principles may underpin the formation and interpretation of gestures, the discussion has shown how a frame-based account may integrate related concepts such as scenes, experiential domains, contiguity (indexicality), similarity (iconicity), and habit/conventionality (symbolicity). Under this unified view, these different concepts provide an insightful lens onto various experientially grounded processes of metonymic motivation that tend to pragmatically induce, in one way or another, not only the forms and functions, but also the habit-driven processes of patterning and schematization that are discernable in gesture. We also saw gestural evidence for the claim that metonymy is experientially more basic than metaphor and hence often feeds into correlated metaphoric processes.

How metonymy plays out in signed language could only be briefly touched upon here in comparison to its role in co-speech gesture. We can preliminarily conclude that metonymic processes typically apply on-the-fly and from scratch in gestures, whose forms and potential meanings are highly context-dependent and not as strongly stabilized as they are in signed languages. However, what I am suggesting here is that the set of metonymic principles discussed in this paper seem to generally operate in visuo-kinetic signs, thus engendering similarly principled ways of forming embodied signs, as well as guiding inferential processes that are implied in their interpretation. Exactly how these metonymic mechanisms systematically compare and differ in gesture and signed language, including gestures occurring within signed discourse, needs to be established through future research across languages, modalities, and discourse genres. Empirical investigation into how metonymic processes are conditioned by interacting experiential, physical, cognitive, cross-modal, modality-specific, discourse, interactional, and cultural forces will no doubt further our understanding of the complex dynamics of multimodal face-to-face interaction.

## Author Contributions

The author confirms being the sole contributor of this work and has approved it for publication.

## Conflict of Interest Statement

The author declares that the research was conducted in the absence of any commercial or financial relationships that could be construed as a potential conflict of interest.

## Footnotes

1 According to Peirce (1960, p. 157), “icons have qualities which resemble those of the objects they represent, and they excite analogous sensations in the mind.” The term ‘Object’ here encompasses existing and imagined entities, persons, actions, etc. For both the gesturer and the interpreter, gestural icons thus rely on a *perceived* similarity between the gestural from and what they are signify.

2 In indexical signs, the relation between sign and object is based on contiguity, according to Peirce, that is, on a factual (physical or causal) connection between a gestural form and what it evokes: “An index is a sign that denotes its object by virtue of being really affected by that object” (Peirce, 1960, p. 143). See also Table 1.

3 The line of research on which this paper builds (Mittelberg, 2006, 2008, 2013, 2014, 2017a; Mittelberg and Waugh, 2009, 2014) combines traditional semiotic frameworks that are not exclusively based on language – notably the works of Jakobson (1956, 1972) and Peirce (1960) – with embodied approaches to language, cognition, and interaction (e.g., Fillmore, 1982; Johnson, 1987; Lakoff, 1987; Goldberg, 1995; Gibbs, 2006; Dancygier and Sweetser, 2014). While the previous work focused on semantic and pragmatic functions of metonymy in gesture, this paper is the first account that integrates related aspects of embodied grammaticalization, multimodal constructions, and lexemes in signed languages.

4 An ethics approval was not required as per applicable guidelines and regulations. Written informed consent was obtained from all participants. For all figures appearing in this paper, written informed consent was obtained from the participants for publication of the images.

5 Langacker (2009, p. 48) defines *active zones* as follows: “An entity’s active zone, with respect to a profiled relationship, is that facet of it which most directly and crucially participates in that relationship.”

6 In sign language, *body partitioning* (Dudis, 2004) also draws on the metonymic organization and affordances of the signer’s body to adopt and combine different viewpoints on a given scene.

7 See Mittelberg and Joue (2017) on gestural *source actions* as metonymic bases of metaphoric processes.

8 Another way to express such a relation is to say that a detail is profiled against the backdrop of a cultural model (Cienki, 1998) or an idealized cognitive model (Lakoff, 1987).

9 For examples of highly abstract complex frame structures, see Mittelberg (2017a: 223ff.).

10 See also Calbris (2011: 78ff.) on *body-focused gestures* and Mittelberg and Waugh (2014) on *body part indices*.

11 See Mittelberg and Waugh (2014) for a typology of predominantly indexical or predominantly iconic gestures, metonymic chains triggered by gestures, and a continuum spanning varying degrees of metonymic proximity.

12 Pointing gestures (e.g., McNeill, 1992; Haviland, 2000; Kendon, 2004; Fricke, 2007) are also commonly observed gestural practices, but limits of space do not allow them to be included in the present discussion.

13 For a brain-imaging study on perceived conventionality in co-speech gestures see Wolf et al. (2017).
